# Mercury concentration in the eggs of four Canadian Arctic-breeding shorebirds not predicted based on their population statuses

**DOI:** 10.1186/2193-1801-2-567

**Published:** 2013-10-26

**Authors:** Meagan McCloskey, Stacey Robinson, Paul A Smith, Mark Forbes

**Affiliations:** Institute of Environmental Science, Carleton University, 1125 Colonel By, Ottawa, ON K1S 5B6 Canada; National Wildlife Research Centre, Environment Canada, 1125 Colonel By Dr., Ottawa, ON K1A 0H3 Canada; Department of Biology, Carleton University, 1125 Colonel By, Ottawa, ON K1S 5B6 Canada

**Keywords:** Mercury, Methylmercury, Shorebird, Egg, Arctic, Population status

## Abstract

Methylmercury is a toxic form of mercury which persists in food webs for long periods of time and biomagnifies up successive trophic levels. Shorebirds breeding in the Arctic are exposed to methylmercury, derived from both natural and anthropogenic sources, when they ingest their invertebrate prey. Populations of many shorebird species are believed to be declining and one hypothesis for these declines is that they are due to detrimental effects of contaminants, including methylmercury. To test this hypothesis, we assessed mercury contamination in eggs of four Canadian Arctic-breeding shorebird species: black-bellied plover (*Pluvialis squatarola*), ruddy turnstone (*Arenaria interpres*), semipalmated plover (*Charadrius semipalmatus*) and white-rumped sandpiper (*Calidris fuscicollis*). Black-bellied plovers and ruddy turnstones are declining in the western hemisphere, whereas white-rumped sandpipers and semipalmated plovers have stable or slightly increasing populations. We found no relationship between egg mercury concentration and population trend for these four shorebird species. Intraspecific variation in mercury concentration was high. Notably, the mercury concentrations were much higher than levels found in a previous study of eggs of the same shorebird species from this same site, suggesting that mercury contamination may be subject to substantial inter-annual variation in the Canadian Arctic food web.

Mercury contamination of the environment is an issue of increasing concern, and studies of the toxic effects on humans and wildlife have become more common (e.g., Albers et al. 2007; Evers et al. 2008; Heinz et al. 2009). The methylated form, methylmercury, is highly toxic, persistent and bioaccumulates in food webs; the top predators generally experience higher levels of methylmercury contamination than species in lower trophic levels (Atwell et al. 1998; Morel et al. 1998; Wiener et al. 2003).

Mercury can enter the environment from natural or anthropogenic sources. Volcanic eruptions, forest fires, and surface water evasion account for some of the natural releases of mercury to the environment (Jasinski 1995; Pirrone et al. 2010). Pirrone et al. (2010) estimate that almost 70% of the world’s mercury emissions are the result of natural sources. Sources of anthropogenic mercury emissions include fossil fuel combustion, metal production and gold mining (Jasinski 1995; Pacyna et al. 2006). Contamination of the natural environment by mercury sources is of concern, particularly in the Arctic ecosystem, which tends to act as a sink for mercury (Ariya et al. 2004).

Mercury is deposited in Arctic ecosystems through oceanic and atmospheric transport (Mason et al. 1994). Gaseous mercury has a long residence time in the atmosphere (~1 year) allowing it to be transported long distances before its deposition on land or ocean surfaces (Mason et al. 1994). Through a phenomenon known as Arctic mercury depletion events, relatively large amounts of gaseous mercury are deposited from the atmosphere into the Arctic ecosystem each spring following polar sunrise (Schroeder et al. 1998).

Mercury deposited from the atmosphere into Arctic lakes can become methylated in anoxic waters by sulphate-reducing bacteria (Morel et al. 1998). It is in this organic form that it can enter the food web by ingestion and bioaccumulate from the lowest trophic level up to the highest (Poissant et al. 2008). Atwell et al. (1998) found that total mercury biomagnified at a rate of approximately 20% per trophic position in an Arctic food web. These findings are supported by numerous observations of higher trophic level organisms, such as birds and mammals, containing elevated concentrations of mercury in their bodies (Braune et al. 2005). In addition, there is the possibility that rising temperatures from climate change might increase the production of methylmercury in Arctic lakes and may extend the season during which it is produced (Stern et al. 2012).

Arctic shorebirds occupy an intermediate trophic position, feeding mainly on terrestrial and aquatic invertebrates. Shorebirds typically arrive on their breeding grounds with minimal fat and have to quickly replenish their energy stores to breed (Klaassen et al. 2001; Morrison and Hobson 2004). The methylmercury taken up by shorebirds can be passed from the female’s blood to her eggs through a process known as depuration (Peterson and Ellarson 1976). Since there is a demonstrated strong positive relationship between female blood mercury and egg mercury (Evers et al. 2003), it is likely that the mercury content of shorebird eggs reflects the contamination level of the local Arctic environment.

High levels of mercury in the eggs of various bird species have been associated with lethal and sublethal effects on the offspring, including reduced hatching success, egg shell thinning and maladaptive chick behaviour (Heinz and Hoffman 2003; Heinz et al. 2009). Mercury contamination of Arctic shorebirds has been measured by sampling eggs (Becker et al. 1992; Mattig et al. 2000), feathers, and other tissues (Hui 1998; Hargreaves et al. 2010,2011); however, lab studies to determine the toxicological effects of mercury on shorebirds are scarce (e.g., Heinz et al. 2009).

Over the past few decades a number of studies have shown some shorebird populations declining throughout North America (Morrison et al. 2001; Morrison et al. 2006; Andres et al. 2012; Ross et al. 2012). Possible reasons for these declines, including loss of coastal stopover habitats to human development, have been suggested but it is difficult to determine causation. The shorebird conservation community in the Western Hemisphere have suggested that the deleterious effects of contaminants may be a contributor to the declines (Butler et al. 2004), but few quantitative data are available to assess this hypothesis. Given the prevalence of mercury in the Arctic environment, mercury contamination of shorebird eggs should be evaluated as one potential contributor to shorebird population declines. In this study, we examined the concentration of mercury in the eggs of four shorebird species that breed at East Bay, Nunavut: black-bellied plover (*Pluvialis squatarola*), ruddy turnstone (*Arenaria interpres*), semipalmated plover (*Charadrius semipalmatus*) and white-rumped sandpiper (*Calidris fuscicollis*). Andres et al. (2012) and Morrison et al. (2006) report that the black-bellied plover and the ruddy turnstone have experienced population declines in North America since the 1970s, based primarily on counts from migratory stopovers. In contrast, white-rumped sandpipers have increased in abundance since the 1970s based on surveys of the fall migration in North America (Andres et al. 2012). The semipalmated plover population is considered to be stable, based on counts from migration surveys and at low-Arctic breeding sites (Andres et al. 2012). These population trends are imprecise because of the challenge of monitoring these wide ranging, migratory species, however, these represent the best estimates of trends for these species, generated using the most comprehensive survey data available (e.g., the International Shorebird Survey, Atlantic Canada Shorebird Survey and the Ontario Shorebird Survey; Andres et al. 2012). Here, we investigate egg mercury concentrations in the above four shorebird species to determine if declining species have higher egg mercury concentration. An additional objective of this study is to establish current mercury levels in eggs and to compare these with the few historical data available (e.g., Hargreaves et al. (2011) did a similar study in 2008), in order to document the magnitude of inter-annual variation in egg mercury levels in the Arctic. Our samples are small and drawn from a single site, and this coupled with the challenges of contaminant studies on wild populations in the field mean that our conclusions are tentative. However, these results help in setting the current baseline, and provide some evidence of patterns in mercury over time in Arctic-breeding shorebirds.

## Materials and methods

### Study area and sampling

**Figure 1:**
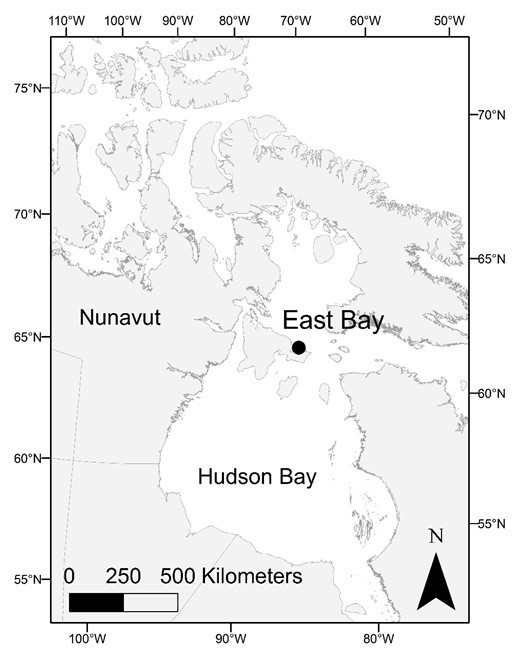
**Map showing the study site at East Bay Mainland Migratory Bird Sanctuary.**

Eggs were collected from active shorebird nests during a nest searching and monitoring effort at East Bay Migratory Bird Sanctuary (63° 59′N 81° 40′W; Figure 1), during the months of June-July 2011. These eggs were collected with approval from Environment Canada Animal Care Committee (EC-PN-11-017) and under a collection permit from the Department of Environment (2011-029). The study area increases in elevation from the coast, with six different identifiable habitat types: intertidal zone, moss carpet, scrub willow, dry heath, sedge meadow, and gravel ridge (Smith et al. 2007). The shorebirds included in this study nest in moderate numbers in each of these habitats.

Five eggs each were collected from the nests of black-bellied plover*,* semipalmated plover*,* ruddy turnstone, and white-rumped sandpiper, with only one egg being taken from each nest. Shorebirds typically lay a clutch of four eggs, so when the nest was found the eggs were labelled according to the number of eggs present in the nest (i.e., “1” meaning first laid egg, “1 of 2” meaning two eggs of unknown order found in the nest, and so on). The eggs were collected once the clutch was complete, to minimize the likelihood of nest abandonment. An attempt was made to collect the first-laid egg. However, in only one instance did we find a nest with a single egg given the difficulty of finding nests during laying (Smith et al. 2009). When the nest initiation date was not observed directly, we used the egg flotation method, which provides an estimate of initiation date ± 4 days in most cases (Liebezeit et al. 2007). After collection, eggs were stored in foam and kept at approximately 4°C for up to 35 days in the field before being transported back to our laboratory at Environment Canada’s National Wildlife Research Centre (NWRC) in Ottawa, Canada.

### Sample preparation

The egg contents were emptied into chemically clean glass jars and stored for 50 days at - 40°C before being analysed for total mercury. The egg contents were prepared for total mercury analysis in the Specimen Preparation Laboratory at the NWRC. For each egg, the entire contents (i.e. yolk and albumen) were homogenized using an Ultra-Turrax homogenizer and then 2 g of the homogenate was collected for the total mercury analysis. These 2 g homogenates were freeze-dried for up to 72 hours.

The total mercury values were reported on a dry mass (dm) basis. The mean percent moisture (%M) of the samples was 73.7 ± 1.2%.

### Mercury analysis

The egg homogenates were analysed for total mercury in the Metals Toxicology Laboratory at the NWRC using the Milestone DMA-80 direct mercury analyzer (Shelton, CT, USA). Total mercury was used as a surrogate measure of the more toxic methylmercury (MeHg), because methylmercury has been found to make up the majority (>98%) of the total mercury found in bird eggs (Scheuhammer et al. 2001; Evers et al. 2003).

Prior to and during the total mercury analysis, quality control measures were taken to ensure that the DMA-80 was functioning properly. These included blank analyses, analyses of standard reference materials and duplicate sample analyses of egg homogenates. The standard reference materials used were NIST freeze-dried oyster tissue (OT1556b), NRCC lobster hepatopancreas (TORT-2) and dogfish liver (DOLT-3). The mean (± S.D.) % recovery for each of the standard reference materials and the mean (± S.D.) % Relative Standard Deviation (standard deviation/mean × 100) for the sample duplicates were reported.

The recovery of mercury in the standard reference materials was from 87 - 106%, and all confidence limits bounded 100% (Table 1). Analysis of duplicates of two of the egg samples showed high analytical precision (% RSD = 1.2 ± 0.45).

**Table 1:** **Summary of quality control results for the analysis of total mercury, showing means (± S.D.) for % recovery and % relative standard deviation (% RSD)**

Quality assurance sample	n	% recovery	% RSD
Oyster tissue 1566b	2	87 ± 0.19	
TORT-2	2	106 ± 0.02	
DOLT-3	4	101 ± 0.02	
Duplicate analysis	2		1.2 ± 0.45

### Statistical analysis

A one-way analysis of variance (ANOVA) was used to determine if there was a significant difference in the mean total mercury concentrations between the species of shorebirds. Data did not differ significantly from a normal distribution (Shapiro-Wilk W test, *W* = 0.92, *P* = 0.12) and a Levene’s test indicated no heterogeneity in variances (*W* = 1.22, *P* = 0.33). Egg mercury concentrations are reported in μg/g dry mass (dm) and the mean (± S.E.) for each species are provided. JMP version 4 (JMP 1989-2007) was used for statistical analysis and statistical significance was determined at p ≤ 0.05.

## Results

### Total mercury

**Figure 2:**
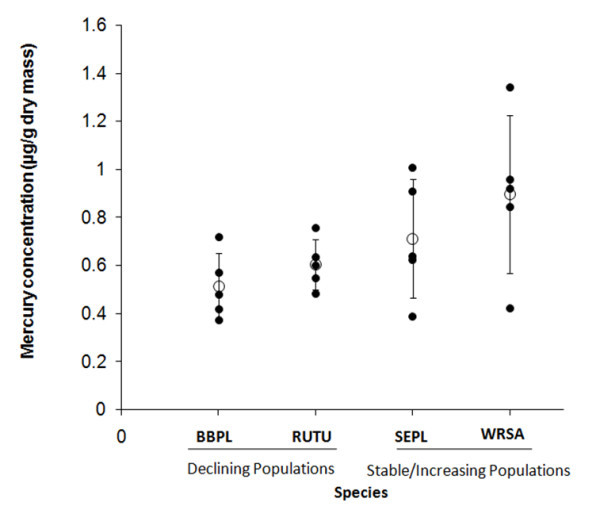
**Total mercury concentration in the eggs (total n=20) of four shorebird species.** BBPL = black bellied plover, RUTU = ruddy turnstone, SEPL = semipalmated plover and WRSA = white-rumped sandpiper. Individual egg values are represented by • and the mean for each species by ο. Error bars show standard deviation. The status of the species population is shown under the species abbreviation.

Total mercury was found in detectable concentrations in all of the shorebird eggs collected for this study (Figure 2; *n* = 5 eggs per species). The total mercury concentration for the shorebird eggs collected ranged from 0.37 μg/g dm in a black-bellied plover egg to 1.34 μg/g dm in a white-rumped sandpiper egg. The white-rumped sandpiper egg that contained the highest mercury concentration of all the eggs collected (1.34 μg/g dm; Figure 2) was known to be the first laid egg of the clutch, otherwise, the positions of the eggs in the laying order of the clutch varied.

There were no significant differences in egg mercury concentrations among the four shorebird species (*F*_*3,16*_ = 2.76; P = 0.08). The total mercury concentrations in the black-bellied plover eggs were generally lower than those found in white-rumped sandpiper eggs (Figure 2). The semipalmated plover and white-rumped sandpiper egg mercury concentrations tended towards higher intraspecific variance (coefficient of variation, CV = 0.35 for semipalmated plover; CV = 0.37 for white-rumped sandpiper) than the black-bellied plover and ruddy turnstone (CV = 0.27 for black-bellied plover; CV = 0.17 for ruddy turnstone), although these differences were not significant in the test of homogeneity of variance.

## Discussion

### Interspecific variation in egg mercury concentrations

As indicated, we did not find any significant differences among species in egg mercury concentration, and therefore no relationship between mercury concentration and population status for these four species of shorebirds. We predicted that mercury concentrations in the eggs of species with declining population trends (i.e., black-bellied plovers and ruddy turnstones) would be higher than those species with stable or increasing population trends (i.e., semipalmated plovers and white-rumped sandpipers). Interestingly, the white-rumped sandpiper (having a stable population trend) had the highest individual egg mercury concentrations of all samples (Figure 2). These findings provide no evidence for mercury contamination of eggs as an obvious explanation for declining populations of shorebirds; however, our samples are small and drawn from a single site, and inter- and intra-specific differences need to be considered when conducting a study of contaminants.

### Interspecific differences in embryo sensitivity to mercury

Avian species differ in embryo and adult sensitivity to methylmercury (Heinz et al. 2009). Mercury concentrations of ~1 μg/g wm (wet mass) were harmful to mallard duck embryos in a study using mercury feeding (Heinz and Hoffman 2003). Heinz et al. (2009) tested the embryos of 26 species of birds and found lethal effects (based on 50% embryo mortality) of injected methylmercury at concentrations ranging from 0.12 μg/g to 2.42 μg/g wm, with some species being overall more sensitive than others. All four shorebird species in this study had mean egg mercury concentrations within this range (mean wm: black-bellied plover = 0.134 μg/g, ruddy turnstone = 0.159 μg/g, semipalmated plover = 0.189 μg/g, white-rumped sandpiper = 0.236 μg/g), suggesting that lethal or sublethal effects are possible, but species specific studies are needed to determine thresholds for toxicity.

One should also be cautious when comparing field results to studies of injected methylmercury. Injected methylmercury is considered much more embryo-toxic than maternally-derived methylmercury because, when injected, the mercury is unbound which allows it to more readily come into contact with the membrane around the embryo (Heinz et al. 2006).

The possibility of shorebirds being less sensitive to mercury than other bird taxa cannot be ruled out. One of the species in the Heinz et al. (2009) study, the American avocet (*Recurvirostra americana;* a shorebird) was found to have a very high median lethal concentration (4.33 μg/g wm; based on a small sample size), suggesting that this species’ embryos are less sensitive to mercury. While taxonomically close, American avocets occupy different wintering (Mexico and Florida) and breeding grounds (central US and south Canada; Robinson et al. 1997) than the four shorebird species in our study and are known to be exposed to high levels of contamination from mercury in their salt pond foraging habitats (Ackerman et al. 2007).

The egg mercury concentrations found in our study are similar to concentrations found in European shorebirds from the Wadden Sea (Becker and Dittman 2010), or among failed snowy plover (*Charadrius alexandrinus*) eggs in California (0.13 – 0.96 μg/g dw; Schwarzbach et al. 2005). Also, similar concentrations to what we present here were observed among long-tailed duck (0.34 – 1.56 μg/g dw) and common eider eggs (0.25 – 1.84 μg/g dw) collected in 2008 from the Canadian Arctic (Akearok et al. 2010).

### Effect of laying order

Female birds tend to have lower concentrations of mercury in their bodies than males likely because they can depurate mercury from their bodies to their eggs (Peterson and Ellarson 1976; Goede and Wolterbeek 1994; Robinson et al. 2012). Egg mercury levels tend to decrease throughout the laying of a clutch, with the first-laid eggs having the highest concentration of mercury and later-laid eggs having less mercury (Becker 1992; Kennamer et al. 2005; Akearok et al. 2010). These previous studies have demonstrated this pattern for terns, gulls and waterfowl (common eiders (*Somateria mollissima*), wood ducks (*Aix sponsa*), and long-tailed ducks (*Clangula hyemalis*)). The extent to which this applies to shorebirds is not entirely known, but Becker (1992) showed that mercury declined, although non-significantly, with laying order in oystercatchers. We could not describe patterns of mercury concentration in relation to laying order because almost all nests were found with near to complete or complete clutches; finding shorebird nests with incomplete clutches is challenging because birds attend the nest only intermittently prior to clutch completion (Norton 1972).

### Foraging habits, arrival time and mercury

The timing of nest initiation may be playing a role in egg mercury concentration. Arctic shorebirds generally use an “income” breeding strategy, where they obtain nutrients needed for egg production from the local breeding grounds (Klaassen et al. 2001; Morrison and Hobson 2004). However, Yohannes et al. (2010) found that sandpipers nesting in the Alaskan Arctic used a mixture of capital and income breeding strategies, depending on their arrival time. Earlier arriving females tended to use stored resources (i.e., from staging areas) for reproduction, whereas later arriving females used more resources directly from the breeding grounds (Yohannes et al. 2010).

At East Bay, timing of arrival of birds to the study area varies little across species (typically <1 week; Smith et al. 2010). Timing of breeding, in contrast, varies by two weeks or more across species. It is possible that females that initiated clutches later had foraged for longer on the breeding grounds, which could affect their mercury contamination compared to early-nesting females. This disparity would be particularly evident if the staging grounds and breeding grounds differ in mercury contamination, because egg mercury concentrations reflect blood mercury levels of the nesting females (Evers et al. 2003).

Observations at East Bay 1999–2007 suggest that ruddy turnstones nest early relative to the other three species, and white-rumped sandpipers nest late and over a longer period (Smith et al. 2010). The elevated (albeit, non-significant) levels of mercury in the eggs of white-rumped sandpipers relative to ruddy turnstones, and the great variability in concentrations for white-rumped sandpipers are consistent with an effect of amount time spent foraging on the breeding grounds. In the future, testing for an effect of nest initiation date on egg mercury concentration (with a larger sample size) could provide some insight into how pre-breeding activities (e.g., foraging) affect mercury dynamics.

### Inter-annual variation in mercury in arctic shorebirds?

The four shorebird species in this study appear to have elevated egg mercury levels when compared to an earlier study (i.e., Hargreaves et al. 2010,2011). The eggs used by Hargreaves et al. (2010,2011) were collected in 2008 from the same four species at the same study site (East Bay). Mean total egg mercury concentrations for each species in this study were at least three times greater than the results from Hargreaves et al. (2011), and the ranges of egg mercury levels found in each study did not overlap. A similar pattern of contamination was observed in Hargreaves et al. (2011) where black-bellied plovers and ruddy turnstones had the lowest egg mercury concentrations. However, in contrast to our study, they observed the highest egg mercury concentrations among the semipalmated plovers, rather than the white-rumped sandpipers (Hargreaves et al. 2011).

This striking difference in egg mercury concentration between years should be interpreted cautiously, as Hargreaves et al. (2011) used inductively coupled plasma mass spectrometry (ICP-MS) with nitric acid digestion for the mercury analysis, whereas this study used the Direct Mercury Analyzer. ICP-MS analyses are known to underestimate true mercury concentrations; when routine procedures are followed for analyses of multiple contaminants (i.e., nitric acid digestion), recovery of mercury in standard reference materials is frequently below 75% (Jian et al. 2000). While methodological differences might explain some of the discrepancy seen across years, we feel that it’s an unlikely explanation for the three-fold differences observed.

Another possibility is that the elevated mercury concentrations in the more recently collected eggs were due to atmospheric mercury cycling. Analysis of long term atmospheric mercury trends at a monitoring site in the Russian Arctic (Amderma) indicated a three-fold increase in the number of Arctic mercury depletion events (AMDEs) between 2008 and 2011 (Pankratov et al. 2013); these AMDEs provide an input for mercury into the Arctic ecosystem, but whether or not this same pattern was observed in the Canadian Arctic is unknown.

The true magnitude and cause of this apparent substantial increase in egg mercury concentration since 2008 is unknown, and might simply be a comparison of two anomalous years. However, given the magnitude of the increase, it merits further study through additional sampling.

## Summary and conclusions

We found no differences in egg mercury concentrations between the species with declining numbers (black-bellied plovers, ruddy turnstones) and the species with stable or increasing numbers (white-rumped sandpipers, semipalmated plovers). These results do not support mercury contamination as a primary contributor to shorebird declines in North America, but ours is not a definitive test. Our sample is small and drawn from a single site, and studies of contaminants in the wild are extremely complex undertakings, with many variables to consider. It is possible that detrimental population-level effects of mercury are not showing up in the shorebird embryonic stage; mercury contamination may be affecting shorebirds at some other stage in their life history, such as at overwintering sites or feeding during migration stopovers. In nature, organisms can be exposed to a toxic “cocktail” consisting of multiple contaminants, possibly acting antagonistically or synergistically with one another depending on life history stage, as mercury and selenium sometimes do (as in mallard ducks; Heinz and Hoffman 1998).

A notable finding of our study is the large differences in egg mercury concentrations for these four shorebird species between 2008 (Hargreaves et al. 2010,2011) and our sampling in 2011. This finding is worrisome and current levels are within ranges observed to cause deleterious effects in other studies. The possibility remains that this difference reflects high variability of mercury concentrations among years rather than a true temporal trend, but even if a product of high variability, this result illustrates the importance of having a program to monitor contaminants in shorebirds of the North. Such a program should attempt to include multiple sites and contaminants to create a broader picture of contamination of shorebirds and their food webs in the Arctic, and also include annual monitoring to document interannual variation.
